# Massive upper gastrointestinal bleeding from a pancreatic pseudocyst rupture: a case report

**DOI:** 10.4076/1757-1626-2-6793

**Published:** 2009-08-17

**Authors:** Gianluca Donatini, Pietro Iacconi, Carmine De Bartolomeis, Chiara Iacconi, Claudio Caldarelli, Davide Caramella, Massimo Chiarugi, Paolo Miccoli

**Affiliations:** 1 Department of Surgery, University of PisaVia Roma 67, 56100 Pisa, PIItaly; 2 Department of Radiology, University of PisaVia Roma 67, 56100 Pisa, PIItaly

## Abstract

**Introduction:**

Bleeding from pancreatic pseudocyst’s rupture into adjacent organs is a rare, but potentially fatal, complication of chronic pancreatitis requiring quick management. Timing of the rupture is unpredictable; early diagnosis and correct management is essential in preventing the bleeding.

**Case presentation:**

We describe the case of a 53 years old male patient successfully treated with emergency surgery for massive hematemesis due to a rupture of a bleeding pseudocyst into the stomach. Patient underwent emergency laparotomy and suture of the bleeding vessel. At 5 years follow-up patient is in healthy condition.

**Conclusion:**

This case shows to surgeons that pancreatic pseudocyst cannot be managed strictly with one rule and prompt surgical treatment is mandatory in case of haemodinamic instability.

## Introduction

Pancreatic pseudocyst is a common finding in patients with previous acute or chronic pancreatitis, developing respectively in up to 15% and 40% of these groups of patients [1]. Rupture of the pseudocyst can result in massive bleeding, with a high mortality rate even for those patients whom undergone treatment. Although it is a quite rare evenience, haemorragic pseudocyst, involving the splenic artery, gastroduodenal and pancreaticoduodenal artery has been described, it should be suspected in all patients with a history of alcoholism, chronic pancreatitis, recurrent abdominal pain and previous gastrointestinal bleeding and promptly treated [2-4].

This report detail a case of sudden haemorragic complication of pancreatic pseudocyst and its emergency surgical management.

## Case presentation

**Figure 1.**
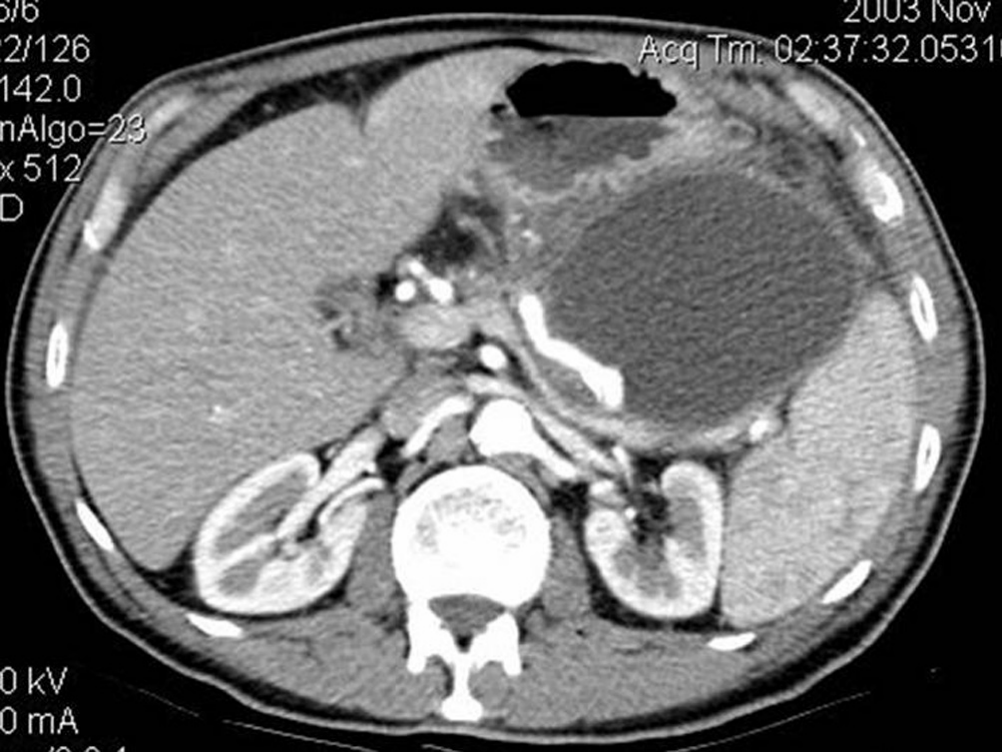
Patient at admission: arterial phase CT shows normal splenic artery immediately behind a large pancreatic pseudocyst.

**Figure 2.**
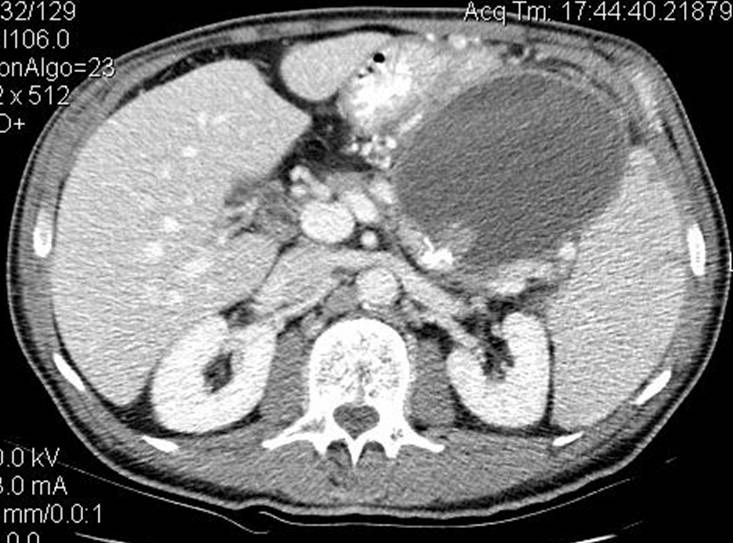
Venous phase CT shows a dubious new finding suggesting a minimal vascular abnormality within the pseudocyst.

**Figure 3.**
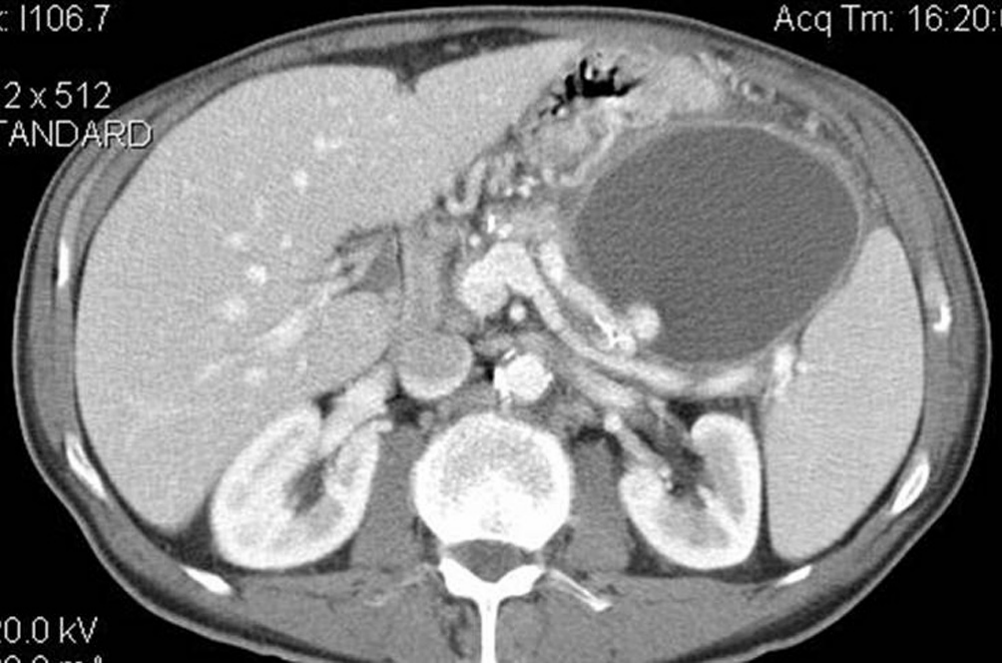
Arterial phase CT shows arterial enhancement pattern of the vascular lesion. Supected diagnosis: splenic artery pseudoaneurysm.

A 53-years Italian, white Caucasian, old male former alcoholic (stop drinking since 11 years) with known chronic pancreatitis came at our Department for mild abdominal pain, anorexia and bowel obstruction. Physical examination was unremarkable except for a mesogastric tenderness. Laboratory findings revealed an increase value of Amilase (148 mg/dl), with slightly increase levels of bilirubin (2.1 mg/dl) and transaminase (AST 76, ALT 82). A CT scan revealed a 10 × 10 × 7 cm tail cystic esofitic lesion of the pancreatic tail in a pattern of chronic pancreatitis (Figure 1). Patients was immediately treated with Somatostatin, fasting and intravenous feeding, with reduction of amylase levels within normal limits in 12 day. MRI examination 48 h after CT scan revealed normal findings of the biliary tract with presence in enhanced signal on the lower part of the cyst suggestive of a blood clot. A further CT scan confirmed the presence of the clot and revealed small multi-infarctual areas within the spleen (Figure 2). Due to medical pre-existent condition (PTCA 3 years before, platelet inhibitor drugs - Ticlopidine) and in a picture of haemodynamic stability, angiographic examination was postponed and CT scan survey (1 scan per week) was done during hospital stay, to assess any evolution within the pseudocyst. Further CT scan revealed an increase of the pseudocyst with enlarged diameter of 13 × 12 × 10 with findings of recent intracystic bleeding (Figure 3). Blood sample examinations anyway did not reveal decrease in haemoglobin or in haematocritus levels.

Ecographic evaluation coupled with CT scan did not diagnose any fluid collection within the abdomen. A CT scan performed 4 weeks from the first diagnosis of acute on chronic pancreatitis, the day before hospital discharge, revealed a reduction of diameters (10 × 10 × 10 cm) and disappearing of infarcted areas within the spleen if compared with the previous one. Few hours after the examination patient presented hypotension, sweating, diaphoresis, massive hematemesis and subsequent haemorrhagic shock.

Emergency laparotomy was performed. Surgical exploration of the pseudocyst was performed through an anterior gastrotomy, revealing a bleeding coming directly from the main trunk of the splenic artery; after adequate surgical vessel isolation the defect was sutured by means of not reabsorbable stitches (4/0 prolene).

**Figure 4.**
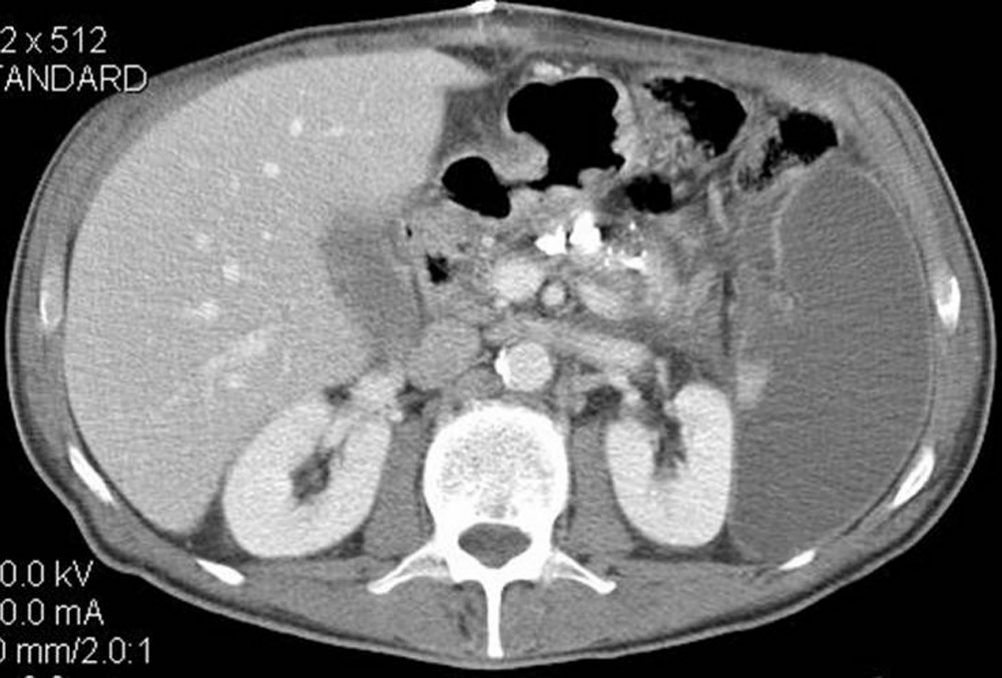
Two weeks post-operative CT scan: in the venous phase the splenic artery is no longer visible; there is hypodensity of the spleen due to reduced vascular supply. Follow-up examinations demonstrated healing of the spleen.

Post-operative course was uneventful. A color Doppler flow of splancic vessels performed 2 weeks after surgery demonstrated normal flow within all arteries with little reduced flow in part of the branches at the splenic hilum. CT scan examination revealed splenic diffuse hypodensity due to reduced blood flow (Figure 4). Patient was then discharged at post-operative day 15th. CT scan and angiographic evaluation performed in the last 5 years did not demonstrated any recurrence of bleeding or new pseudocyst within the pancreas.

## Discussion

Pancreatic pseudocysts are common conditions following both acute and chronic pancreatitis or pancreatic trauma [5]. Different techniques for surgical drainage have been described and may be used for those fluid collections that have no regressed or increased in size after 6 weeks from detection [6]. In patients hospitalized for acute pancreatitis in a pattern of chronic disease, CT scan survey allow the surgeon to treat with drainage a pseudocyst progressively increasing in diameter or to identify an arterial bleeding to be embolized. Pseudocyst presentation may vary widely ranging from mild nausea with anorexia, to gastric outlet obstruction, abdominal pain and jaundice. Bleeding is a rare complication, due to Elastase action on the arterial wall, involving less than 5% of patients although carrying a mortality rate greater than 40% and potentially affecting all the splanchnic vessels [3,4,7,8]. Angiographic scan is the gold standard examination to assess any source of bleeding of the pseudocyst or pseudoaneurysm af splanchnic artery, but as in the case presented, concomitant co-morbidity (Ticlopidine assumption) may lengthen the application of this item. Moreover on it’s not possible to predict which patient is going to bleed and when just on the diameter of the pseudocyst.

## Conclusion

This case reminds to surgeons that pancreatic pseudocyst cannot be managed strictly with one rule [5], but strategy may vary in each different patients following clinical evolution. Prompt surgical treatment is mandatory in case of haemodynamic instability.

## Abbreviations

ALT: Alanine transaminase
AST: Aspartate aminotransferase
CT: Computed tomography scan
MRI: Magnetic resonance imaging
PTCA: Percutaneous transluminal coronary angioplasty
